# Single-Fraction Radiotherapy for CD30^+^ Lymphoproliferative Disorders

**DOI:** 10.1155/2015/629587

**Published:** 2015-10-04

**Authors:** Michelle S. Gentile, Maria Estela Martinez-Escala, Tarita O. Thomas, Joan Guitart, Steven Rosen, Timothy Kuzel, Bharat B. Mittal

**Affiliations:** ^1^Department of Radiation Oncology, Northwestern University, Robert H. Lurie Comprehensive Cancer Center of Northwestern University, Chicago, IL 60611, USA; ^2^Department of Dermatology, Northwestern University, Robert H. Lurie Comprehensive Cancer Center of Northwestern University, Chicago, IL 60611, USA; ^3^Department of Radiation Oncology, Loyola University, Chicago, IL 60153, USA; ^4^Division of Medical Oncology, Department of Medicine, Northwestern University, Robert H. Lurie Comprehensive Cancer Center of Northwestern University, Chicago, IL 60611, USA

## Abstract

*Objectives*. CD30^+^ lymphoproliferative disorder is a rare variant of cutaneous T-cell lymphoma. Sustained complete response following first-line treatments is rare. This retrospective review evaluates the response of refractory or recurrent lesions to palliative radiation therapy. *Methods*. The records of 6 patients with 12 lesions, treated with radiation therapy, were reviewed. All patients received previous first-line treatments. Patients with clinical and pathological evidence of symptomatic CD30^+^ lymphoproliferative disorder, with no history of other cutaneous T-cell lymphoma variants, and with no prior radiation therapy to the index site were included. *Results*. The median age of patients was 50.5 years (range, 15–83 years). Median size of the treated lesions was 2.5 cm (range, 2–7 cm). Four sites were treated with a single fraction of 750–800 cGy (*n* = 3) and 8 sites were treated with 4000–4500 cGy in 200–250 cGy fractions (*n* = 3). Radiation therapy was administered with electrons and bolus. Median follow-up was 113 months (range, 16–147 months). For all sites, there was 100% complete response with acute grade 1-2 dermatitis. *Conclusions*. For recurrent and symptomatic radiation-naïve CD30^+^ lymphoproliferative disorder lesions, palliative radiation therapy shows excellent response. A single fraction of 750–800 cGy is as effective as a multifractionated course and more convenient.

## 1. Introduction

Primary cutaneous T-cell lymphomas (CTCLs) are relatively rare, with an annual incidence of 7 in 1,000,000 [1]. Primary cutaneous CD30^+^ lymphoproliferative disorders (LPD) represent 25–30% of CTCLs and are the second most common form after mycosis fungoides (MF [2]). CD30^+^ LPD can be divided further into lymphomatoid papulosis (LYP) and primary cutaneous anaplastic large-cell lymphoma (CALCL) with substantial overlap between the two diagnoses resulting in a spectrum of disease. Although molecular markers and genetic rearrangements can be used to aid in diagnosis, histology alone can be insufficient and clinical course is often used to determine diagnosis and treatment [3, 4].

There are five histological subtypes of LYP with A being the most common presentation and B, C, and D resembling MF, CALCL, and CD8^+^ cytotoxic T-cell lymphoma, respectively [4]. There is also a recently described rare angioinvasive variant of LYP designated as histological subtype E [5]. Immunohistochemistry often shows CD30^+^ expression with large pleomorphic or anaplastic T cells. LYP is a chronic indolent disease with recurrent papulonodular lesions that present over a course of years to decades and may spontaneously regress after weeks to months. LYP has an excellent prognosis with a 5-year disease specific survival of 100% [2]. Patients with LYP, however, are at greater risk of second cutaneous or nodal lymphoid malignancies that precede, follow, or are associated with other lymphomas such as MF, cutaneous, or nodal anaplastic large-cell lymphoma and Hodgkin's Lymphoma [3, 6, 7]. It has been described that LYP, Hodgkin's Lymphoma, and CTCL can be derived from a single T-cell clone and a t(8:9) genetic translocation may be involved in the pathogenesis of LYP or its progression to malignant disease [8].

Similar to LYP, CD30^+^ expression is seen in >75% of CALCL cells, which have a large anaplastic, pleomorphic appearance [2]. CALCL presents as rapidly growing solitary or localized nodules that are rarely multifocal, with the appearance of large ulcerating tumors or thick plaques. Spontaneous complete resolution or partial regression is commonly reported in >40% of patients [4]. Skin relapse is common, with extracutaneous dissemination to mainly regional lymph nodes occurring in approximately 10% of patients. 10-year disease specific survival for patients without lymph node involvement is >90% [2].

There have been many therapeutic approaches for LYP including topical steroids, psoralen plus ultraviolet light therapy (PUVA), and low-dose methotrexate, which may show high response rates [9, 10]. CALCL lesions are often treated with radiation therapy (RT) or surgery for localized disease or low-dose methotrexate. In the case of rapidly progressive or extracutaneous disease, treatment is with multiagent doxorubicin-based chemotherapy and more recently brentuximab vedotin [2, 11]. There is often spontaneous complete regression of smaller LYP lesions, but with larger lesions (>1-2 cm), a diagnosis of CALCL is more seriously considered and regression becomes less predictable. Relapse after dose reduction or withdrawal of treatment is at least 40% and often much higher with LYP lesions in particular, and often these patients have lifelong disease with frequent relapse [4]. Due to high relapse rates, maintenance therapy may be used but may be accompanied by long-term complications including a higher incidence of nonmelanoma skin cancer and possible development of hepatic complications from chronic methotrexate use [12]. In addition, misinterpretation of the clinical presentation of CD30^+^ LPD for a more aggressive disease (i.e., lymphoma, melanoma, or carcinoma) and the increased incidence of secondary lymphoid neoplasms in LYP patients have led to treatment with systemic chemotherapy or even bone marrow transplantation [3, 13]. Kempf et al. [4] consensus guidelines for the treatment of CD30^+^ LPD recommend consideration of RT for persistent, larger lesions greater than 2 cm.

Yu et al. [14] have described the treatment of CALCL with RT as an effective treatment modality. Outcomes of LYP patients treated with RT, however, have been sparse and inconsistent. There are no known prospective studies, and much of the published data is anecdotal or from small case studies. Total skin electron beam therapy (TSEBT) and localized fractionated RT have been used with variable response [15–23]. There is limited information on RT details and follow-up [3, 24]. Many of these reports included patients with synchronous or antecedent cutaneous lymphomas making assessment of response of LYP lesions challenging. In addition, there are no studies of single-fraction palliative RT for LYP or CALCL.

Since 1999, we have treated a small series of patients with refractory or recurrent symptomatic CD30^+^ LPD lesions using both multifractionated and single-fraction RT. This retrospective analysis describes the largest series of patients with CD30^+^ LPD treated with localized radiation for palliation.

## 2. Materials and Methods

After approval by the Institutional Review Board, our department records were examined and a comprehensive chart review was performed yielding 6 patients with CD30^+^ LPD who were treated with localized single or multifractionated palliative RT to 12 individual lesions between October 1999 and July 2012. Distinction among CD30^+^ LPD and borderline cases can be challenging; thus patients were carefully selected so that borderline diagnoses were excluded. The European Organization for Research and Treatment of Cancer (EORTC)/International Society for Cutaneous Lymphoma (ISCL)/United States Cutaneous Lymphoma Consortium (USCLC) consensus recommendations on primary cutaneous CD30^+^ LPD were used to confirm the diagnosis of all patients [4]. The TNM staging system for primary cutaneous lymphomas other than MF and Sézary syndrome as proposed by the ISCL/EORTC was retroactively used for staging [25]. Patient and tumor characteristics were assessed at initial consultation. All patients had disease refractory to prior topical and/or systemic treatment and no history of other CTCLs or skin disorders and had not received prior RT to the index site. Date of last follow-up was defined as the last encounter by a radiation oncologist, medical oncologist, or dermatologist where response to the treated lesion had been documented. Patients were seen in follow-up 1 month following treatment and scheduled at 3–6-month intervals thereafter. Death was confirmed by search of public death records. Pathology reports were reviewed with the dermatopathologist in order to determine the immunophenotype and histological type for the six patients (see Figure 2).

Each lesion receiving RT was categorized based on its location. Parameters of RT assessed included total dose, dose per fraction, energy, and bolus thickness. Response was defined in a manner consistent with that put forth by the EORTC/ISCL/USCLC consensus recommendations on primary cutaneous CD30^+^ LPD [4]; a CR was defined as 100% clearance of the skin lesion treated, a partial response (PR) was defined as a reduction in lesion size of more than 50% but less than 100%, and stable disease (SD) was defined as a less than 50% reduction in size of the lesion. Relapse was defined as any disease recurrence in those with CR. All patients had a CR; thus no patient or tumor characteristics were studied for correlation with response. There were no identified relapses.

The RT regimen consisted of 750–800 cGy delivered in a single fraction to 4 lesions or 200–250 cGy delivered in multiple fractions for a total of 4000–4600 cGy to 8 lesions in the earlier years. En face electron technique was used for superficial lesions on flatter surfaces. Electron energy consisted of 10 or 12 MeV. Bolus was used for all of the lesions with a 0.5 or 1 cm thick material in order to increase radiation dosage to the skin. Electron dose was prescribed to the 90–95% isodose line.

## 3. Results

Using the strict criteria described above, 6 patients with 12 localized, CD30^+^ LPD lesions were treated with palliative RT.

This study consisted of 3 female and 3 male patients with a total of 12 lesions. The median age was 50.5 years (range, 15–83 years) at initial time of RT treatment (Table 1). The median diameter of the lesion was 2.5 cm (range, 2–7 cm). All patients had a history of biopsy proven CD30^+^ LPD; six of the 12 lesions had pathological confirmation while the remainder of patients were described as having lesions that waxed and waned or recurrent papulonodular lesions refractory to first-line therapy consistent with their history of CD30^+^ LPD. All lesions continued to progress following first-line or other therapies and were symptomatic and none of the lesions had evidence of spontaneous regression. One patient received oral methotrexate prior to RT and another patient in the earlier years received CHOP chemotherapy prior to RT for a synchronous diagnosis of subcutaneous Non-Hodgkin's Lymphoma. All patients presented with generalized skin involvement consistent with T3 disease [25]. Of the 6 lesions with pathological confirmation, all showed a CD4^+^/CD30^+^ immunophenotype. Three lesions were of type C LYP histology and 1 lesion was of type A LYP histology with the remainder not specified.

**Figure 1:**
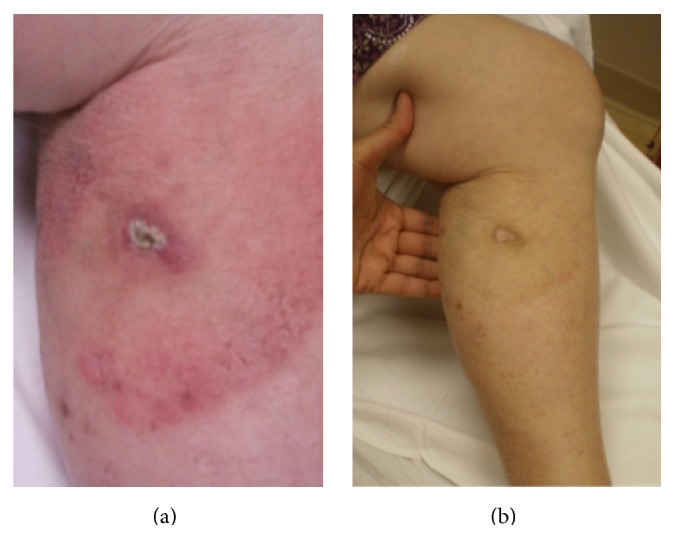
(a) Patient with primary cutaneous CD30^+^ lymphoproliferative disorder (LPD) of the left lower extremity. The gross lesion is a raised nodule with central ulceration and surrounding erythema. (b) The same patient at follow-up visit 8 months after completion of a single fraction of radiation therapy (RT) to 800 cGy. There is no clinical evidence of residual cutaneous lymphoma. All what remains is fibrotic tissue, which continues to fade. There was no evidence of recurrence at the last follow-up 27 months after treatment.

**Figure 2:**
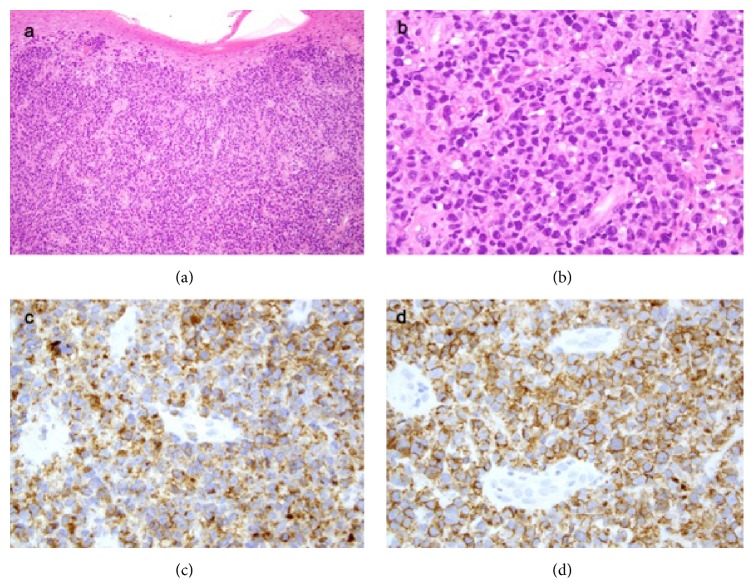
(a) Dense dermal atypical lymphocytic infiltrate with mild epidermotropism. (b) Detail of the atypical lymphocytes arranged in sheets and mitotic figures are identified. Atypical lymphocytes are positive for (c) CD4 and (d) CD30.

All patients had a CR to radiation (Table 2; Figure 1). RT was well tolerated, with the only recorded toxicity being grade 1-2 dermatitis. Median follow-up was 113 months (range, 16–146 months) for the group as a whole. For the patients receiving a single fraction of RT, the median follow-up was 22.5 months (range, 16–37 months). For the patients receiving a multifractionated course of RT in the earlier years, the median follow-up was 131 months (range, 66–146 months). All of the 6 patients were alive with disease at last follow-up with no evidence of relapse at the treatment site.

## 4. Discussion

Cutaneous CD30^+^ LPD is an indolent, recurrent variant of CTCL that has been shown to be radiosensitive. Recent consensus recommendations include surgical excision or RT for larger (defined as >2 cm in diameter) persistent lesions as an alternative approach to waiting for spontaneous regression [4]. In regard to this recommendation, however, there is little recent published evidence as to the clinical efficacy of local radiation or the recommended dose, fractionation scheme, technique, or long-term follow-up, especially with regard to LYP. Diagnosis by histology alone remains challenging and clinical presentation is often important. This small retrospective series represents the largest series to date specifically reporting localized RT outcomes for CD30^+^ LPD using a multifractionated and single-fraction approach.

A critical review of the literature (Table 2) showed that all studies used a multifractionated course of RT for treatment of LYP, with total doses of 8–40 Gy administered through either TSEBT or localized superficial RT. Willemze et al. [15] treated one patient with two separate lesions. The first lesion was treated with TSEBR to a total dose of 40 Gy; the patient experienced a CR but locally recurred within 3 months. A second lesion was treated with localized RT to a total dose of 25 Gy; again the patient initially experienced a CR but locally recurred within 5 months. The patient went on to develop a systemic lymphoma. Sanchez et al. [16] treated 4 of 31 patients with LYP using various radiation therapies including TSEBR to a total dose of 30 Gy and reported no response in 3 of 4 patients. Kaufmann et al. [19] treated 1 of 2 patients with localized RT to a total dose of 35 Gy in 2.0 Gy fractions using 6 MeV electrons and reported a durable CR, although follow-up was not specified. Wilson et al. [18] reported that treatment of 3 of 161 patients with LYP/CTCL with TSEBR to a total dose of 30 Gy resulted in a 3-year DFS of 20%; all patients had relapsed by 4.8 months. J. Breneman and D. Breneman, [17] in an editorial response to this study, shared anecdotal results of 5 patients treated with TSEBR to a total dose of 36 Gy with a CR rate of 80% and no relapse at a minimum follow-up of 12 months. Kaufmann et al. [20] also reported a favorable outcome in one patient treated with localized RT to 30 Gy in 2.0 Gy fractions using 6 MeV electrons with a CR and no recurrence at a follow-up of 45 months. Taken together, results for LYP treated with a multifractionated course of RT resulted in a 69% CR rate, but relapse was approximately 45% at a follow-up of 3-4 months.

In addition, Yu et al. [14] showed a 100% CR rate for 8 patients with CALCL treated with a multifractionated course of RT ranging from 34 to 44 Gy in 2.0 Gy fractions with a median follow-up of 12 months. Other retrospective studies have shown excellent CR rates for CALCL patients; however, data for patients treated with RT alone is lacking, especially with regard to specific RT dose, technique, and long-term follow-up [14].

There have been few studies where a few fractions of low-dose RT were given to treat LYP. Sina and Burnett [22] treated 2 of 5 patients with a course of 6 Gy in 2.0 Gy fractions, resulting in 100% CR rate and no relapse at 14 and 36 months. Scarisbrick et al. [23] treated 2 of 4 patients with 8 Gy in 2.0 Gy fractions; although there was a 50% CR rate, the first patient showed a CR with an additional 8 Gy. The remainder of studies lacked sufficient information on total dose, fractionation scheme, technique, or follow-up [3, 21, 24]. In our study, there was a 100% CR rate, supporting our data for single-fraction RT for palliation of CD30^+^ LPD. Moreover, Thomas et al. [26] have shown a 94.4% CR rate for primarily MF lesions treated with a single fraction of localized palliative RT at a mean follow-up of 41.3 months.

CD30^+^ LPD is more likely to be multifocal, presenting as a recurrent, self-healing papulonodular eruption that often spontaneously resolves without treatment over weeks to months. All patients were referred for treatment of lesions that had not shown spontaneous regression with continued growth following first-line or other therapies and, thus, were concerning for a diagnosis of CD30^+^ LPD. Given that all patients had a durable CR at the RT site with sufficient follow-up, these response rates are likely to be reflective of treatment itself and not due to the spontaneous regression of the lesions. A second criticism may be that, given the overlap of CD30^+^ LPD with CALCL, as well as the tendency toward progression or concurrent MF or Hodgkin's Lymphoma, unambiguous histological diagnosis may be difficult [5, 27–29].

## 5. Conclusion

CD30^+^ LPD is a radiosensitive CTCL variant. In addition to a multifractionated course of RT, a single fraction of 750–800 cGy is effective in inducing a durable CR with minimal acute side effects. Longer follow-up is necessary before conclusions regarding local control can be made especially for patients treated with a single fraction. This study is the largest retrospective series reporting palliative RT dose, technique, treatment outcomes, and long-term follow-up supporting palliative localized RT for symptomatic CD30^+^ LPD refractory or recurrent to other therapies.

## Figures and Tables

**Table 1:** Characteristics of CD30^+^ lymphoproliferative disorder (LPD) patients treated with radiation therapy (RT).

Patient	Sex/age (y) at presentation	Antecedent or synchronous lymphoma	TNM stage^*∗*^ at presentation	Number of lesions treated with RT	Location of lesion	Date of completion of RT	Clinical or pathologic diagnosis	Histological type	Immunophenotype	Prior therapies	Total dose (Gy)/dose per fraction/energy/ bolus/IDL	Response at RT site	LR/follow-up (m)
1	M/48	n/a	T3b	1	Right lower extremity	08/15/11	Path.	n/s	CD4^+^/CD30^+^	Oral/IL methotrexate, topical imiquimod	8/8, 12 MeV e^−^, 1 cm bolus, 90% IDL	CR	N/16

2	M/60	Synchronous subcutaneous NHL	T3b	6	Left groin	12/26/02	Clin.			CHOP × 6c	45/2.5, 10 MeV e^−^, 1 cm bolus, 90% IDL	CR	N/131
					Right femoral region	12/26/02	Clin.				45/2.5, 10 MeV e^−^, 1 cm bolus, 90% IDL	CR	N/131
					Right axilla	12/26/02	Path.	C	CD4^+^/CD30^+^		45/2.5, 10 MeV e^−^, 1 cm bolus, 90% IDL	CR	N/131
					Left axilla	12/26/02	Clin.				45/2.5, 10 MeV e^−^, 1 cm bolus, 90% IDL	CR	N/131
					Right neck	4/7/03	Path.	n/s	CD4^+^/CD30^+^		45/2.5, 10 MeV e^−^, 1 cm bolus, 90% IDL	CR	N/127
					Right elbow	3/01/06	Clin.				45/2.5, 10 MeV e^−^, 1 cm bolus, 90% IDL	CR	N/99

3	F/83	Antecedent HL	T3b	2	Left lateral thigh	11/12/10	Path.	n/s	CD4^+^/CD30^+^	IL methotrexate, IL triamcinolone acetonide	8/8, 10 MeV e^−^, 1 cm bolus, 95% IDL	CR	N/37
					Left calf	09/07/11	Path.	C	CD4^+^/CD30^+^	IL steroids	8/8, 12 MeV e^−^, 1 cm bolus, 90% IDL	CR	N/27

4	F/53	n/a	T3a	1	Upper lip	10/21/99	Path.	A	n/s		42/2, 10 MeV e^−^, 0.5 cm bolus, 90% IDL	CR	N/146

5	F/34	n/a	T3b	1	Left forearm	7/23/12	Path.	C	CD4^+^/CD30^+^	Topical methotrexate, PUVA, triamcinolone acetonide	7.5/7.5, 12 MeV e^−^, 1 cm bolus, 90% IDL	CR	N/18

6	M/15	n/a	T3b	1	Left back	9/28/08	Clin.			RT, prednisone, minocycline	40/2, 12 MeV e^−^, 1 cm bolus, 95% IDL	CR	N/66

^*∗*^Staging per Kim et al., 2007 [25].

HL = Hodgkin's Lymphoma, NHL = Non-Hodgkin's Lymphoma, n/s = not specified, IL = intralesional, e^−^ = electron, IDL = isodose line, CR = complete response, AWD = alive with disease, LR = local recurrence, and N = none.

**Table 2:** Studies including lymphomatoid papulosis (LYP) patients treated with radiation therapy (RT).

Author [ref.]	Study type	Number of LYP patients treated with RT	Number of LYP lesions treated	Associated lymphoma	Locations of treated lesion	TNM stage at presentation^*∗*^	Other treatments	RT details	CR rate	LR	Time to LR (m)	Follow-up (m)
Thomsen and Schmidt [21]	E	1	1	MF	n/s	T3bN0	Topical 5-FU, topical steroids, PUVA	Localized RT	100%	Y	n/s	

Willemze et al. [15]	R	1	2	LCL	n/s			TSEBR, 40 Gy, 4 MeV	100%	Y	3	
					n/s			Localized RT, 25 Gy, 100 kV	100%	Y	5	

Sanchez et al. [16]	R	4		MF	n/s	T3aN0	Topical corticosteroids, photochemotherapy, chlorambucil, prednisone, combination chemotherapy	TSEBR, 30 Gy, 6 MeV e^−^	0%	N		
				MF	n/s	T3aN0	Topical nitrogen mustard	Localized RT	0%	N		
				HL	n/s		Mustine HCL, vincristine, sulfate, prednisone, procarbazine HCL		100%	Y	n/s	
				ML	n/s			Localized RT	0%	N		

Sina and Burnett [22]	R	2	2		Left thigh	T3bN0		Localized RT, 6/2 Gy, 15 kV, 30 mm HVL	100%	N		14
					n/s	T3bN0		Localized RT	100%	N		36

Kaufmann et al. [19]	R	1	2		Right forearm	T1bN0		Localized RT, 35/2–2.5 Gy, 6 MeV e^−^, 0.5 cm bolus, 90% IDL	100%	N		
					Right finger			Localized RT, 30/2 Gy, 6 MeV e^−^	100%	N		

Wilson et al. [18]	R	3			n/s	T1-3Nx	Topical steroids, PUVA, topical nitrogen mustard	TSEBR/6 fields, 36/1 Gy, 6 MeV e^−^, supplemented 20/1 Gy, 120 kV to perineum, soles of feet and 6/2 Gy, 120 kV to apical scalp	100%	Y	4.8; 3-year DFS 20%	44.1 (median)

J. Breneman and D. Breneman [17]	E	5		MF	n/s		n/s; 1 patient received concurrent CHOP	TSEBR/modified Stanford, 36 Gy	80%	N		12, 48, 61, and 70

Christensen et al. [24]	R	6	6		n/s			Localized RT	100%	N		

Cabanillas et al. [3]	R	4		CTCL/MF/ LCL	n/s				0%	Y	n/s	

Kaufmann et al. [20]	R	1	4		Right forearm, left forearm ×3	T1bN0		Localized RT, 30/2 Gy, 6 MeV e^−^, 0.5 cm bolus daily	100%	N		45

Scarisbrick et al. [23]	R	2	2		Left flank	T2aN0		Localized RT, 8/2 Gy, 100 kV	0% (100% with retreatment)	Y	6	12
					Lower abdomen	T2aN0		Localized RT, 8/2 Gy, 100 kV	100%	N		12

^*∗*^Retrospective staging according to Kim et al., 2007 [25].

E = editorial, R = retrospective, MF = mycosis fungoides, LCL = large-cell lymphoma, HL = Hodgkin's Lymphoma, ML = malignant lymphoma NOS, CTCL = cutaneous T-cell lymphoma, PUVA = psoralen and ultraviolet A therapy, n/a = not applicable, n/s = not specified, TSEBR = total skin electron beam radiotherapy, e^−^ = electron, HVL = half value layer, LR = local recurrence, and DFS = disease-free survival.

## Abbreviations

CALCL: Cutaneous anaplastic large-cell lymphoma
CR: Complete response
DFS: Disease-free survival
EORTC: European Organization for Research and Treatment of Cancer
ISCL: International Society for Cutaneous Lymphoma
LPD: Lymphoproliferative disorders
LYP: Lymphomatoid papulosis
MF: Mycosis fungoides
CTCLs: Cutaneous T-cell lymphomas
PR: Partial response
PUVA: Psoralen plus ultraviolet light therapy
RT: Radiation therapy
SD: Stable disease
TSEBT: Total skin electron beam therapy
USCLC: United States Cutaneous Lymphoma Consortium.
